# Evaluation of Cotton Leaf Curl Virus Resistance in BC_1_, BC_2_, and BC_3_ Progenies from an Interspecific Cross between *Gossypium arboreum* and *Gossypium hirsutum*

**DOI:** 10.1371/journal.pone.0111861

**Published:** 2014-11-05

**Authors:** Wajad Nazeer, Abdul Latif Tipu, Saghir Ahmad, Khalid Mahmood, Abid Mahmood, Baoliang Zhou

**Affiliations:** 1 State Key Laboratory of Crop Genetics and Germpalsm Enhancement, MOE Hybrid Cotton R&D Engineering Research Center, Nanjing Agricultural University, Nanjing, Jiangsu Province, China; 2 Cotton Research Station, Multan, Ayub Agricultural Research Institute, Faisalabad, Punjab, Pakistan; 3 Cotton Research Institute, Ayub Agricultural Research Institute, Faisalabad, Punjab, Pakistan; USDA-ARS-SRRC, United States of America

## Abstract

Cotton leaf curl virus disease (CLCuD) is an important constraint to cotton production. The resistance of *G. arboreum* to this devastating disease is well documented. In the present investigation, we explored the possibility of transferring genes for resistance to CLCuD from *G. arboreum* (2n = 26) cv 15-Mollisoni into *G. hirsutum* (2n = 52) cv CRSM-38 through conventional breeding. We investigated the cytology of the BC_1_ to BC_3_ progenies of direct and reciprocal crosses of *G. arboreum* and *G. hirsutum* and evaluated their resistance to CLCuD. The F_1_ progenies were completely resistant to this disease, while a decrease in resistance was observed in all backcross generations. As backcrossing progressed, the disease incidence increased in BC_1_ (1.7–2.0%), BC_2_ (1.8–4.0%), and BC_3_ (4.2–7.0%). However, the disease incidence was much lower than that of the check variety CIM-496, with a CLCuD incidence of 96%. Additionally, the disease incidence percentage was lower in the direct cross 2(*G. arboreum*)×*G. hirsutum* than in that of *G. hirsutum*×*G. arboreum*. Phenotypic resemblance of BC_1_ ∼BC_3_ progenies to *G. arboreum* confirmed the success of cross between the two species. Cytological studies of CLCuD-resistant plants revealed that the frequency of univalents and multivalents was high in BC_1_, with sterile or partially fertile plants, but low in BC_2_ (in both combinations), with shy bearing plants. In BC_3_, most of the plants exhibited normal bearing ability due to the high frequency of chromosome associations (bivalents). The assessment of CLCuD through grafting showed that the BC_1_ to BC_3_ progenies were highly resistant to this disease. Thus, this study successfully demonstrates the possibility of introgressing CLCuD resistance genes from *G. arboreum* to *G. hirsutum*.

## Introduction

Cotton production is biotically constrained by various diseases, which lead to yield instability and reduced seed quality. Cotton leaf curl disease (CLCuD) is a debilitating disease of cotton in Africa, Pakistan, and Northwestern India [1]–[3]. CLCuD is caused by a pathogen complex of a virus and a DNA beta satellite (DNA-β) molecule [4]. There are seven such virus species, all belonging to the *Begomovirus* genus, and DNA-β satellites are associated with CLCuD in these regions [5]–[8].

CLCuD was first recorded in 1967 in the Multan district, Pakistan, on scattered *Gossypium hirsutum* plants [9]–[11], and it has spread rapidly to all cotton growing areas of Pakistan and throughout the Indian subcontinent. Two epidemics of this disease have been observed during the past three decades due to a loss of host-plant resistance in existing cotton varieties [12]–[13].

In Pakistan, an outbreak of CLCuD occurred in the early 1990s. This disease devastated the Pakistani cotton industry, where it caused an estimated yield reduction of 30–35%. Between 1992 and 1997, the economic losses due to CLCuD in Pakistan amounted to approximately 5 billion dollars (US) [14]. Similarly, in the Indian state of Punjab, this disease reduced cotton production by almost 70% in 1998 [15]. Singh et al. [16] observed a reduction of 52.7% in the number of bolls and a reduction of 54.2% in boll weight due to CLCuD, whereas the differences in yield loss between resistant and susceptible cultivars were almost 50% and 85–90%, respectively.

In the late 1990s, several resistant cotton varieties were gradually introduced into the Indo-Pak region, and losses due to the disease diminished [17]–[18]. However, resistance subsequently broke in 2001–2002 [3], [12] due to new strains of CLCuD emerged, and all of the cotton varieties that were previously known resistant to CLCuD, such as LRA-5166, CP-15/2, and Cedex, have become susceptible to CLCuD [6]–[7], [19]–[23]. Symptoms of this disease were also reported in China [24], which is located far from the hot spots of India and Pakistan, and there is great concern that CLCuD could spread from its origin to other cotton growing areas of the world where the disease is not currently present. Plant biologists have attempted to understand the molecular biology of this disease complex to control CLCuD [25], but the tricky nature of the pathogen and the rapid evolution/recombination of these genes have hindered the progress of this research [26]–[28].

In plant breeding, wild relatives have long been studied due to the presence of novel genes [29]–[31], and these wild species have been exploited most often as sources for biotic and abiotic stress resistance [32]. Among the wild species of cotton, especially, desi cotton (*G. arboreum* L.) has built in desirable resistant genes for all kind of *Begomoviruses* associated with CLCuD [33]. Additionally, *G. arboreum* is known to combat various stresses like drought [34]–[35], heat [36], root rot, cotton leaf curl virus [37] and insect pests (bollworms and aphids) [12]. Interspecific hybridization of cotton has been performed with varying degrees of success [21], [38]–[40]. For example, Sacks and Robinson [41] transferred nematode (*Rotylenchulus reniformis*) resistance into tetraploid *G. hirsutum.* Chen et al. [42] and Nazeer et al. [2] employed *Gossypium australe* and *Gossypium stocksii* to introgress some novel genes for drought and CLCuD resistance into *G. hirsutum,* respectively. The interspecific hybridization is quite difficult, especially, between *G. arboreum* and *G. hirsutum*, and some scientists explored bridge lines for introgression of genes form wild species [43].

At present, no single variety of *G. hirsutum* is resistant to CLCuD; however, *G. arboreum* is documented to have resistance against CLCuD [31]. Due to the importance of this disease and significant features of this species, we initiated a project to explore the possibility of successful transferring CLCuD resistance genes from Desi cotton (*G. arboreum*, 2n = 26) into cultivated upland cotton (*G. hirsutum*, 2n = 52) genotypes through conventional hybridization and backcrossing without developing bridging line. In this way maximum desirable donor genes of *G. arboreum* can be transferred into *G. hirsutum* to improve the resistance to CLCuD of the cultivated *G. hirsutum*.

## Materials and Methods

### Plant materials

**Figure 1:**
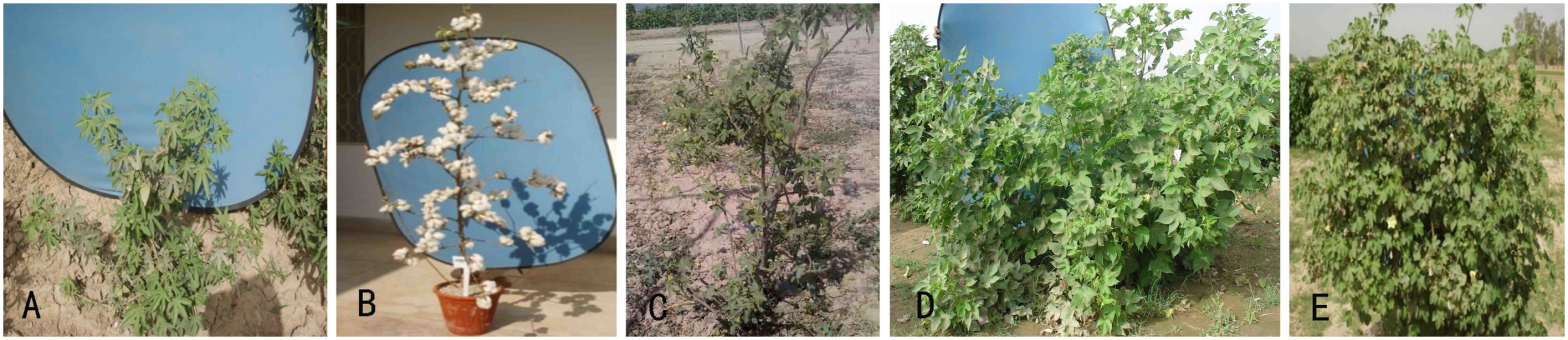
Parents of interspecific hybridization. A. *G. arboreum* cv 15-Mollisoni (2n = 2x = 26); B. *G. hirsutum* cv CRSM-38 (2n = 4x = 52); C. 2(*G. arboreum*) (2n = 4x = 52); D. [2(*G. arboreum*)×*G. hirsutum*] F_1_ (2n = 4x = 52); E. (*G. hirsutum*×*G. arboreum*) F_1_ (2n = 3x = 39).

The plant materials used in this study include *G. hirsutum* cv CRSM-38 (2n = 4x = AADD = 52), *G. arboreum* cv 15-Mollisoni (2n = 2x = AA = 26), and an artificial autotetraploid of *G. arboreum* cv 15-Mollisoni (2n = 4x = 52; Figure 1). The F_1_ CLCuD-resistant progeny involving these parents, which was developed by Ahmad et al. [44], comprising direct cross [2(*G. arboreum*)×G. *hirsutum*] and its reciprocal cross (*G. hirsutum*×*G. arboreum*), was utilized to directly backcross with *G. hirsutum*. The number of F_1_ progenies was increased by cuttings. Thus total number of F_1_ plant progenies for direct and reciprocal cross was 10 and 15, respectively, to generate BC_1_ to BC_2_ generations. CIM-496, a cotton variety highly susceptible to CLCuD, was employed as a standard/control in order to obtain a natural virus inoculum.

### Development of backcross progenies

**Figure 2:**
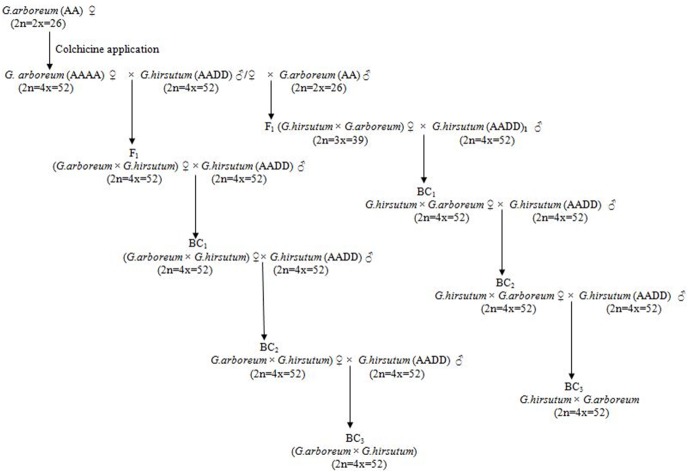
Scheme for the development of the BC_1_ to BC_3_ progenies for interspecific cross 2(*G. arboreum*)×G. *hirsutum* and *G. hirsutum*×*G. arboretum*.

The F_1_ CLCuD-resistant progenies consisting of two cross combinations, 2(*G. arboreum*)×G. *hirsutum* and *G. hirsutum*×*G. arboreum*, were backcrossed with *G. hirsutum* to produce the BC_1_ progenies in 2011. The BC_1_ progenies were planted in the field at Cotton Research Station in Multan, Pakistan in May 2012 and backcrossed with *G. hirsutum* to generate the BC_2_ progenies. These BC_2_ progenies were again backcrossed with *G. hirsutum* cv CRSM-38 to produce the BC_3_ progenies in 2013. One thing should be noticed here that only those normal morphological plant progenies that produce more fruits but no symptoms of CLCuD were selected for backcrossing. The plant progenies that showed even minor spots of CLCuD were rejected to utilize for backcrossing. The scheme for the development of the backcross progenies are shown in Figure 2. Emasculation was carried out in the evening, and emasculated flowers were manually pollinated the next morning.

### Use of plant growth hormones for hybridization

Normally, embryos fail to develop in hybridizations between *G. arboreum* and *G. hirsutum.* This obstacle was overcome by the application of plant hormones such as gibberellic acid (GA_3_) and naphthalene acetic acid. Specifically, 50 mgL^−1^ GA_3_ and 100 mg L^−1^ naphthalene acetic acid were applied to the bases of pedicles 24 hours after pollination for 3 consecutive days to reduce embryo and boll shedding. The number of cross boll sets was counted, and the bolls were picked at harvest time.

### Cross fertility studies

Fertility studies for BC_1_ to BC_3_ progenies of 2(*G. arboreum*)×G. *hirsutum* and *G. hirsutum*×*G. arboreum* were measured in term of cross boll setting and their germination percentage by given formula:
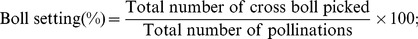


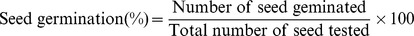



### Morphological characteristics

Observations of growth habit, stem color, leaf texture, leaf shape, leaf hairiness, bracteole size, corolla color, petal spots, the position of the staminal column, anther color, and dehiscence in the parents, as well as in BC_1_ to BC_3_, were recorded. The phenotypic resemblance of BC_1_ to BC_3_ progenies to *G. arboreum* having desirable traits with good resistance to CLCuD will be helpful for selection of introgression progenies.

### Cytological studies

Morphological normal plants producing more fruits were selected from BC_1_ to BC_3_ progenies for cytological studies. Young buds of BC_1_ to BC_3_ plants, along with those of the parents, were collected and fixed in Carnoy’s solution at 8 to 9 am and preserved in 70% ethanol after 24 h. Three to four anthers were squashed on a slide with a drop of 2.5% acetocarmine solution to examine the pollen mother cells (PMCs). Chromosomal configurations such as univalent (I’s), bivalents (II’s), trivalents (III’s), quadrivalents (IV’s), and division stage were examined under a Labomed microscope, and photographs were also taken using a camera mounted on a Labomed microscope.

### Maintenance of virus inoculum and screening for CLCuD

Artificial inoculation techniques is not available for CLCuD, therefore, the only way to study the response of cotton germplasm is to expose the introgression progenies to high inoculum pressure by planting in natural hot spots [45], so sick plot technique was used to arrange spreader plants among BC introgression lines. In this sick plot technique, we planted susceptible variety CIM-496 after each two rows of CLCuD resistant lines to encourage uniform spread of the disease. Planting of BC_1_∼BC_3_ progenies was done after 3^rd^ week of May for the three seasons i.e. 2011–2013. Sowing was done manually and row to row (75 cm) and plant to plant (30 cm) distance was maintained. Row length for each genotype was 450cm and plot size was variable depending upon the seed availability.

### Phenotypic assessment of BC_1_ to BC_3_ progenies against CLCuD

The resistance of the BC_1_ to BC_3_ progenies against CLCuD was assessed under natural field conditions using an inoculum of CIM-496 at Cotton Research Station in Multan, Pakistan which is hot spot of CLCuD. Data for CLCuD were recorded following the rating system described in Table 1 to calculate the severity index (SI), percent disease index (%, DI), and disease reaction. Individual plant ratings for each genotype were added and means were calculated to generate the corresponding SI. The DI was calculated using the following formula:
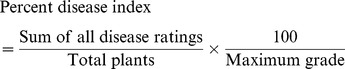



**Table 1:** Disease rating (symptom rating) scale for evaluation of cotton leaf curl virus disease.

Disease index (%)	Severity grade	Symptoms	Remarks
0	0	No Symptoms	Resistant
1–20	1	Thickening of only secondary and tertiary veins.	Highly tolerant
21–30	2	Thickening of secondary and primary (mid rib) veins.	Tolerant
31–50	3	Vein thickening (V.T), leaf curling (L.C) or enation (E) or both.	Susceptible
>50	4	Stunting along with vein thickening leaf curling/enation.	Highly susceptible

The percent disease tolerance (PDT) was calculated by selecting a minimum of 100 plants on a diagonal from one corner to the other, and diseased plants were counted to determine the PDT using the formula:
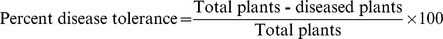



Data regarding the latent period, number of virus-infected plants, disease incidence percentage, disease severity index, infection type, and disease reaction were recorded.

### Inoculation of CLCuD through grafting

A petiole and rootstock from CIM-496 were used to transfer virus inoculum into healthy plants. Two grafting techniques, i.e., approach grafting and petiole grafting, were employed to confirm the resistance against CLCuD in BC_1_ to BC_3_ plants. For approach grafting, the resistant plants of the BC_1_, BC_2_, and BC_3_ progenies were used as scions, whereas virus-susceptible *G. hirsutum* plants were used as stock. For petiole grafting, young petioles from CLCuD-infected plants were selected and inserted into the test plants. Two infected petioles were also grafted onto the same plant to introduce additional virus inoculum. The following data were recorded: grafting success, infectivity, latent period, infection type, disease severity index, and disease incidence percentage at 40 and 70 days after grafting (DAG).

## Results

### Cross fertility studies

**Figure 3:**
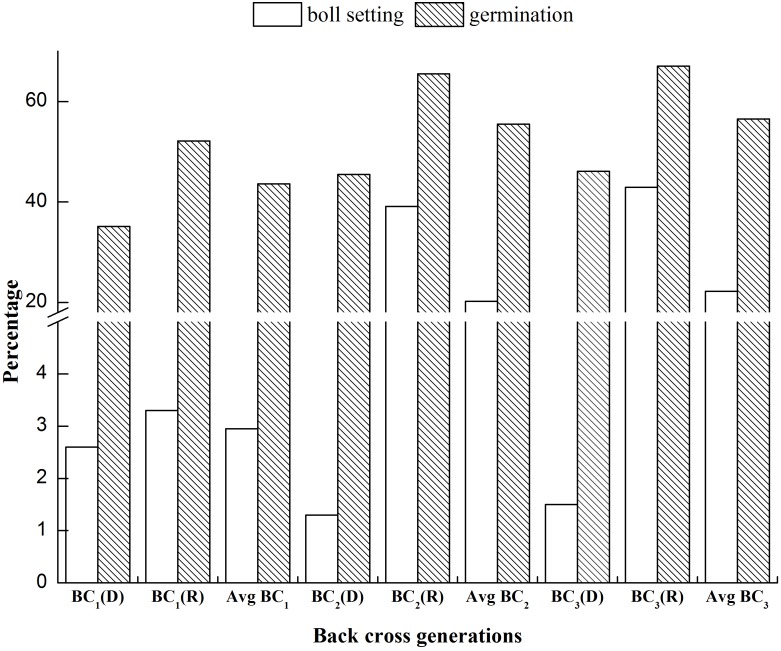
Advancement of boll setting and germination (%) across different generations (BC_1_ to BC_3_). D = Direct cross [2(*G. arboreum*)×*G. hirsutum*]; R = Reciprocal cross [*G. hirsutum*×*G. arboreum*].

Examination of the cross ability of BC_1_ to BC_3_ of the combination 2(*G. arboreum*)×*G. hirsutum* and *G. hirsutum*×*G. arboreum* revealed that the maximum percentage of boll set (42.9%) and germination (67.0%) were observed in the BC_3_ (*G. hirsutum*×*G. arboreum*) progenies (Table 2). Minimum boll setting (1.3%) was recorded in the cross BC_2_ [2(*G. arboreum*)×*G. hirsutum*]. A minimum percentage of viable seeds (35.1%) were obtained in BC_1_ [2(*G. arboreum*)×*G. hirsutum*]. The boll setting and germination (%) gradually increased from BC_1_ to BC_3_ (Figure 3).

**Table 2:** Fertility studies of interspecific hybrid between *G. arboreum* and *G. hirsutum* to produce the BC_1_ to BC_3_ progenies.

Parentage	Year	No. ofplants*	No. ofpollinations	No. of bollspicked	Boll setting(%)	No. of seedobtained	No. of seedgerminated	Germination(%)
BC_1_ [2(*G. arboreum*)×*G. hirsutum*]	2009–11	15	12890	338	2.6	57	20	35.1
BC_1_ (*G. hirsutum*×*G. arboreum*)	2009–11	12	8144	265	3.3	48	25	52.1
BC_2_ [2(*G. arboreum*)×*G. hir*sutum]	2012	14	1495	19	1.3	22	11	45.5
BC_2_ (*G. hirsutum*×*G. arboreum*)	2012	155	299	117	39.1	519	340	65.5
BC_3_ [2(*G. arboreum*)×*G. hirsutum*]	2013	12	980	15	1.5	52	24	46.1
BC_3_ (*G. hirsutum*×*G. arboreum*)	2013	225	7263	3123	42.9	412	276	67.0

*Number of plants used for pollination and recording data.

### Morphological studies

**Figure 4:**
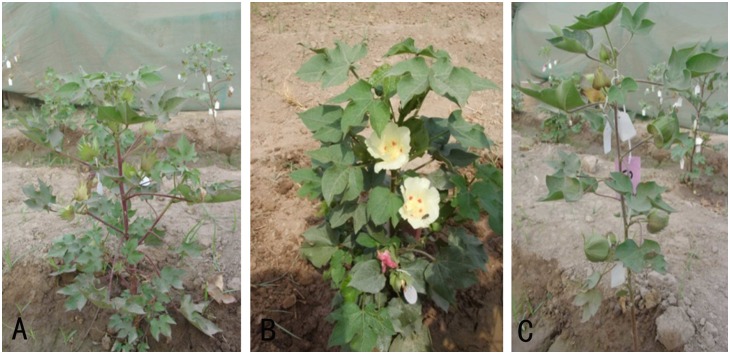
Hybrid Progenies of [2(*G. arboreum*)×*G. hirsutum*]. A = BC_1_; B = BC_2_; C = BC_3_.

Examination of the morphological characteristics of the parents and BC_1_ to BC_3_ of 2(*G. arboreum*)×*G. hirsutum* revealed that in BC_1_ to BC_3_, leaf hairiness, flower size, corolla color, petal spots, and pollen color were segregated for the male and female parents. Stem color, leaf lobation, flower size, corolla color, petal number, petal size, anther dehiscence, and pollen color of BC_1_ to BC_3_ were similar to those of the female parents. Stem hairiness, gossypol glands, leaf size, leaf hairiness, leaf texture, petiole length, and bracteole number and size were dominant characters of the male parents. Bracteole dentation, petiole size, petal spots, and position of the staminal column of BC_1_ to BC_3_ were intermediate between those of both parents (Table 3). The hybrid plant progenies of BC_1_ to BC_3_ of 2(*G. arboreum*)×*G. hirsutum* are shown in Figure 4.

**Table 3:** Morphological characteristics of parents and the BC_1_ to BC_3_ progenies from the cross 2(*G. arboreum*)×*G. hirsutum.*

Morphological characteristic	2(*G. arboreum*)	*G. hirsutum*	BC_1_	BC_2_	BC_3_
**Stem characteristics**					
Stem color	Greenishbrown	Green	Brown	Brown	Brown
Stem hairiness	Profuselyhairy	Hairy	Hairy	Hairy	Hairy
Black glands	Dense	Sparse	Sparse	Sparse	Sparse
**Leaf characteristics**					
Leaf color	Darkgreen	Green	Light/dark green	Light/dark green	Light/darkgreen
Leaf size(cm)	Medium(7.3×9.9)	Large(10×14)	Small/Large(7.0×8.0 cm)/(9.0×11.0 cm)	Small/Large(7.1×8.3)/(9.2×10.8)	Medium/Large(7.6×8.9)/(9.6×11.4)
Leaf hairiness	Profuselyhairy	Hairy	Hairy/profuselyhairy	Hairy/profuselyhairy	Hairy/profuselyhairy
Leaf lobation	3–5narrrow,deeplobed	3–5 broad,shallow lobed	3–5 broadlobed	3–5 broadlobed	3–5 broadlobed
Leaf texture	Thick,Leathery	Herbaceous	Herbaceous	Herbaceous	Herbaceous
Petiole length(cm)	Medium(4.4)	Long(8.8)	Long (7.3)	Long (7.2)	Long (7.5.0)
**Boll characteristics**					
Bracteole numberand size (cm)	2–3, large(3.0×2.6),united atbase	3 large(3.3×1.8)	3 Large (3.0×2.3)	3 Large(3.3×2.2)	3 Large(3.1×2.4)
Bracteole dentation	Entire	5–11,deep narrow	4–9 medium	3–10 medium	3–11 medium
**Flower characteristics**					
Flower size	Medium	Large	Medium	Medium	Medium
Pedicel size (cm)	Long (1.7)	Long (1.2)	Long (1.3)	medium(1.0)	medium(0.9)
Calyx	5 sepalforming acup withwavymargins	5 sepalsforming acup withteeth	5 sepal forminga cup withwavy margins	5 sepal forminga cup withwavymargins	5 sepal forminga cup withwavymargins
Corolla color	Lightyellow	Creamy	Creamy/lightyellow	Creamy/ligtyellow	Creamy/lightyellow
Petal numberand size (cm)	5, medium,(3.0×2.6)	5, large(4.6×4.5)	5, medium(3.5×4.1)	5, medium(3.4×4.2)	5, medium(3.3×3.5)
Petal spot	Darkpink	Absent	Present/absent	Present/absent	Present/absent
Position ofstaminalcolumand size (cm)	Short (0.4)	Long (2.0)	Medium (1.5)	Medium (1.7)	Medium (1.6)
Antherdehiscence	Partial	Normal	Partial	Normal	Normal
Pollencolor	Lightyellow	Creamy	Yellow	Creamy/lightyellow	Creamy/lightyellow
Pistilsize (cm)	Long (2.5)	Long (2.9)	Long (2.6)	Long (2.8)	Long (2.9)

**Figure 5:**
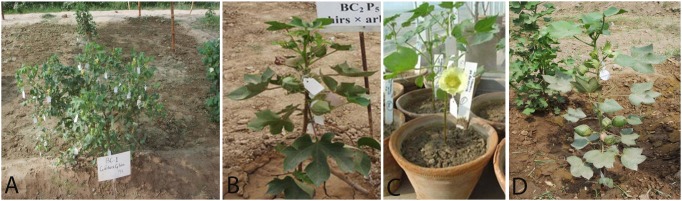
Hybrid Progenies of (*G. hirsutum*×*G. arboreum*). A = BC_1_; B, C = BC_2_; D = BC_3_.

An analysis of the morphological characteristics of the parents and BC_1_ to BC_3_ of *G. hirsutum*×*G. arboreum* revealed that in BC_1_ to BC_3_, gossypol, bracteole number and size, and pistil size was dominant characters of the female parents. Leaf size, leaf lobation, corolla color, petal spots, and pollen color were the dominant characteristics of the male parents. Stem color and hairiness, leaf texture, bracteole number and size, and position of staminal column of BC_1_ to BC_3_ were intermediate between those of both parents, while leaf hairiness was segregated (Table 4). The hybrid plant progenies of BC_1_ to BC_3_ of *G. hirsutum*×*G. arboreum* are shown in Figure 5. Morphological characteristics particularly leaf texture, leaf size, bracteole size, corolla color and petal spots from *G. arboreum* into BC_1_ to BC_3_ progenies of both crosses helped for selection of plant progenies that have some resemblance of *G. arboreum* and also showed CLCuD resistance.

**Table 4:** Morphological characteristics of parents and the BC_1_ to BC_3_ progenies from the cross *G. hirsutum*×*G. arboretum.*

Morphological characteristics	*G. hirsutum*	*G. arboreum*	BC_1_	BC_2_	BC_3_
**Stem characteristcs**					
Stem color	Green	Green	Green	Green	Green
Stem hairiness	Hairy	Hairy	Hairy	Hairy	Hairy
Black glands	Sparse	Sparse/dense	Sparse	Sparse	Sparse
**Leaf characteristcs**					
Leaf color	Green	Green	Green	Green/dark green	Green/dark green
Leaf size(cm)	large(10.0×14.0)	Small/medium(6.0×8.3)	Medium (7.1×7.9)	medium (7.0×8.4)	medium (7.3×8.3)
Leaf lobation	3–5 broad,shallow lobed	3–5 narrrow,deep lobed	3–5 medium lobed	3–5 broad lobed	3–5 broad lobed
Leaf texture	Herbaceous	Herbaceous	Herbaceous	Herbaceous	Herbaceous
Leaf hairiness	Hairy	Hairy/profuselyhairy	Hairy	Hairy/profuselyhairy	Hairy/profuselyhairy
Petiolelength (cm)	Long (8.8)	Medium (4.4)	Long (7.2)	Long (7.5)	Long (7.4)
**Boll characteristcs**					
Bracteole number and size (cm)	3 large (3.3×1.8)	3, small(2.7×2.1),united at base	3, large (3.0×2.6)	3, large (3.0×2.0)	3, large (3.2×2.2)
Bracteole dentation	3–7, superficial	5–11, deepnarrow	4–9, superficial	3–11,superficial	3–9, superficial
**Flower characteristcs**					
Flower size	Large	Small	Medium	Medium	Medium/large
Pedicel size (cm)	Long (1.2)	Long (1.2)	Long (1.1)	Long (1.3)	Long (1.2)
Calyx	5 sepalforming acup withteeth	5 sepalsforminga cup withwavy margins	5 sepalsforming a cupwith wavymargins	5 sepals forminga cup with wavymargins	5 sepals forming acup with wavymargins
Corolla color	Creamy	Yellow	Creamy/LightYellow	Creamy/lightyellow	Creamy/lightyellow
Petal numberand size (cm)	5, large,(4.6×4.5)	5, small(2.6×2.5)	5, large,(4.5×4.4)	5, large,(4.4×4.6)	5, large,(4.6×4.4)
Petalspot	Absent	Lightpink	Present/absent	Present/absent	Present/absent
Position ofstaminalcolumand size (cm)	long (2.0)	small (1.0)	Medium (1.5)	Medium (1.5)	Medium (1.6)
Antherdehiscence	Normal	Normal	Partial/normal	Normal	Normal
Pollencolor	Creamy	Yellow	Creamy/lightYellow	Creamy/lightYellow	Creamy/lightYellow
Pistilsize (cm)	Long (2.9)	Small (2.1)	Long (3.1)	Long (2.9)	Long (3.2)

### Cytological studies

#### Meiosis in parents

**Figure 6:**
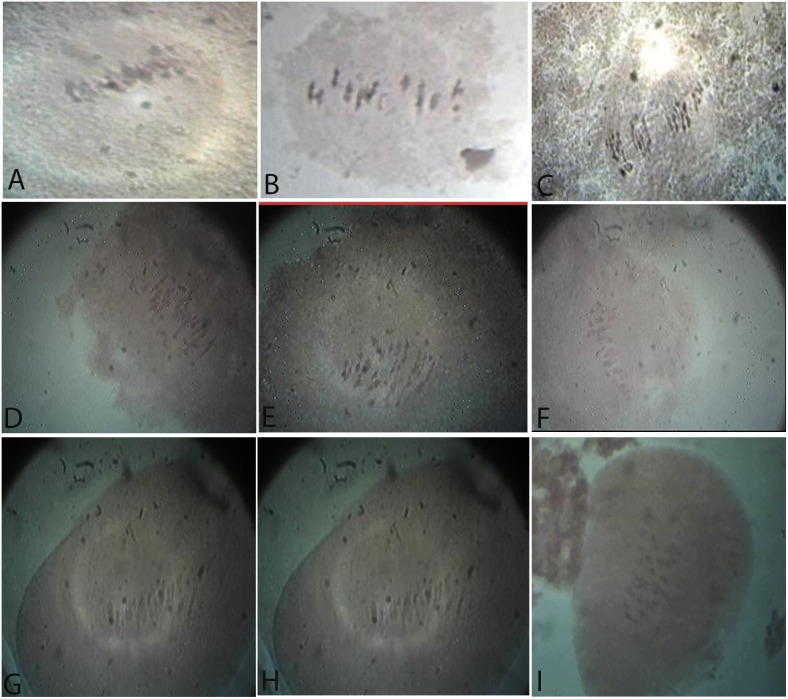
Chromosome configurations in PMCs at Metaphase-I of meiosis. A. *G. hirsutum,* 26 II’s; B. *G. arboreum,* 13 II’s; C. 2(*G. arboreum*), 2 I’s+23 II’s+1 IV; D. [2(*G. arboreum*)×*G. hirsutum*] BC_1_, 6 I’s+21 II’s+1 IV; E. [*G. hirsutum*×*G. arboreum*] BC_1_, 2 I’s+23 II’s+1 IV; F. [2(*G. arboreum*)×*G. hirsutum*] BC_2_, 2 I’s+23 II’s+1 IV; G. [*G. hirsutum*×*G. arboreum*] BC_2_, 3 I’s+21 II’s+1 III+1 IV; H. [2(*G. arboreum*)×*G. hirsutum*] BC_3_, 2 I’s+25 II’s; I. [*G. hirsutum*×*G. arboreum*] BC_3_, 26 II’s.

The course of meiosis was examined in the *G. hirsutum* and *G. arboreum* parents. In these species, the reduction division was normal, with regular pairing of chromosomes. The number of bivalents in *G. hirsutum* and *G. arboreum* at Metaphase-I was 26 and 13, respectively (Figures 6A and 6B). The disjunction of the chromosomes was normal at Anaphase-I. The meiotic behavior in the artificial autotetraploid of *G. arboreum* parent showed two I’s, 23 II’s, and one IV (Figure 6C).

**Meiosis in BC_1_.** **[2(** ***G. arboreum*)×*G. hirsutum*].**The progenies of this combination comprised 15 plants; only seven normal morphological plants with better boll setting were studied cytologically. Cytological studies at Metaphase-I revealed that there were six I’s, 21 II’s, and one IV’s (Figure 6D). The number of I’s, II’s, III’s, and IV’s for 76 PMCs ranged from 5–12, 18–22, 0–1, and 0–1, respectively, for a total of 52 chromosomes (Table 5), while the average number of I’s, II’s, III’s, and IV’s was 8.2, 20.4, 0.2, and 0.7, respectively. A few lagging chromosomes were also observed at Anaphase-I. The high frequency of univalents (5–12) and multivalents (0–1) caused meiotic disturbance; the plants were partially fertile/sterile.

**Table 5:** Cytological comparison of BC_1_ to BC_3_ plants from an interspecific cross between *G. arboreum* and *G. hirsutum*.

Cross congifuration	Plant number	PMC	I’s	II’s	III’s	IV’s	Total
**Chromosomal configuarion for BC_1_**							
2(*G. arboreum*)×*G. hirsutum*	P2	12	5	20	1	1	52
^ //^	P3	10	6	21	0	1	52
^ //^	P4	8	6	21	0	1	52
^ //^	P9	10	8	20	0	1	52
^ //^	P11	15	8	22	0	0	52
^ //^	P13	11	12	20	0	0	52
^ //^	P4	10	12	18	0	1	52
^ ^	Range		5–12	18–22	0–1	0–1	
^ ^	Average of 76 cells		8.2	20.4	0.2	0.7	
*G. hirsutum*×*G. arboreum*	P1	5	2	25	0	0	52
^ //^	P2	12	1	22	1	1	52
^ //^	P5	8	5	20	1	1	52
^ //^	P9	10	2	23	0	1	52
^ //^	P10	6	2	25	0	0	52
^ //^	P11	12	2	25	0	0	52
^ ^	Range		1–5	20–25	0–1	0–1	
^ ^	Average of 53 cells		2.2	23.2	0.4	0.6	
**Chromosomal configuarion for BC_2_**							
2(*G. arboreum*)×*G. hirsutum*	P3	10	2	25	0	0	52
^ //^	P4	15	4	24	0	0	52
^ //^	P7	8	2	25	0	0	52
^ //^	P10	12	2	23	0	1	52
^ //^	P11-(1)	15	4	24	0	0	52
^ ^	Range		2–4	23–25	0	0–1	
^ ^	Average of 60 cells		3.0	24.1	0	0.2	
(*G. hirsutum*×*G. arboreum*)	P1(16)	15	1	24	1	0	52
	P2	10	3	23	1	0	52
		12	2	25	0	0	52
	P4 (1)	10	2	23	0	1	52
^ //^	P5(3)	10	2	25	0	0	52
^ //^		8	4	22	0	1	52
^ //^	P5(14)	5	3	21	1	1	52
^ //^	P7(15)	5	3	23	1	0	52
^ ^		10	2	23	0	1	52
^ //^		10	2	23	0	1	52
^ ^	P9(16)	8	4	22	0	1	52
^ //^	P13(17)	7	3	21	1	1	52
^ ^	Range		1–4	21–25	0–1	0–1	
^ ^	Average of 110 cells		2.4	23.2	0.4	0.5	
**Chromosomal configuarion for BC_3_**							
2(*G. arboreum)*×*G. hirsutum*	P1	15	2	25	0	0	52
	P2	20	2	25	0	0	52
	Range		2	25	0	0	
	Average of 35 cells		2	25	0	0	
*G. hirsutum*×*G. arboreum*	P1	20	0	26	0	0	52
^ //^	P14	20	0	26	0	0	52
^ ^	Range		0	26	0	0	
^ ^	Average of 40 cells		0	26	0	0	

***G. hirsutum*** **×** ***G. arboretum*** **.** The plant progenies of this combination comprised 12 plants; cytological studies were conducted on six normal morphological plants with better boll setting. The cytological configuration at Metaphase-I of the BC_1_ plants revealed two I’s, 23 II’s, and one IV’s (Figure 6E). In the 53 PMCs of these hybrid plants, there were 1–5 I’s, 20–25 II’s, and 0–1 III’s and IV’s, for a total of 52 chromosomes (Table 5), while the average number of I’s, II’s, III’s, and IV’s for 53 PMCs was 2.2, 23.2, 0.4, and 0.6, respectively. Although multivalent association was observed, the high frequency of bivalents (20–25) caused these plants to be fertile or partially fertile.

**Meiosis in BC_2_.** **[2(** ***G. arboreum*)×*G. hirsutum*].**These plant progenies consisted of 14 plants; only five normal morphological plants with better boll setting were studied cytologically. The chromosomal conformation at Metaphase-I was two I’s+23 II’s+1 IV’s (Figure 6F). A study of 60 PMCs revealed 2–4 I’s, 23–25 II’s, and 0–1 IV’s, for a total of 52 chromosomes (Table 5), while the average number of I’s, II’s, and IV’s for 60 PMCs was 3.0, 24.1, and 0.2, respectively. Trivalents were not observed in these plants. The low frequency of uni- and multi-valents, as well as the high frequency of chromosome association (23–25 II’s), caused the plants to be fertile but shy bearing.

***G. hirsutum*** **×** ***G. arboretum.*** The plant progenies of this combination comprised 161 plants; only 10 normal morphological plants with better boll setting were studied cytologically. The chromosomal constitution at Metaphase-I revealed 3 I’s+21 II’s+1 III’s+1 IV’s (Figure 6G). However, in 110 PMCs, there were 1–4 I’s, 21–25 II’s, and 0–1 III’s and IV’s, for a total of 52 chromosomes (Table 5), and the average number of I’s, II’s, III’s, and IV’s was 2.4, 23.2, 0.4, and 0.5, respectively. Low frequencies of univalents (1–4) and multivalents (0–1), as well as high frequencies of bivalents (21–25), were observed. The plants were fertile. A few shy bearing plants were also observed.

**Meiosis in BC_3_.** **[2(** ***G. arboreum*** **)×*G. hirsutum*** **].** The plant progenies consisted of 12 plants. A total of 35 PMCs were sampled from two plants for microscopic studies. Metaphase-I of these PMCs showed 2 I’s+25 II’s (Figure 6H). The average range of these PMCs revealed that there were 2 I’s and 25 II’s, for a total of 52 chromosomes (Table 5); the plants were fertile.

***G. hirsutum×G. arboretum.*** The plant progenies consisted of 225 plants. The chromosome pairing was normal (26 II’s) in most of the PMCs (Figure 6I). The average number of chromosomes among 40 PMCs exhibited normal disjunction (Table 5); the plants were fertile.

### Testing of BC_1_ to BC_3_ progenies against CLCuD through grafting

**Figure 7:**
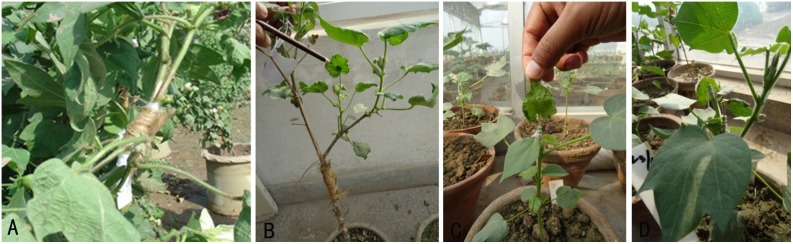
Testing of CLCuD through grafting. A and B. Cleft grafts; C = Single petiole grafts; D = Double petiole grafts.

The resistance/susceptibility of the plants was confirmed through petiole and approach grafting, as indicated in Figure 7, and only resistant plants were used for backcrossing to produce the next generation. Grafting for BC_1_ to BC_3_ hybrid plants of 2(*G. arboreum*)**×** *G. hirsutum* and *G. hirsutum* **×** *G. arboreum* was carried out under greenhouse conditions as well as in the natural field. All plants from both crosses [2(*G. arboreum*)**×** *G. hirsutum* and *G. hirsutum* **×** *G. arboreum*] showed 100% infectivity and grafting success (Table 6). Plants of susceptible variety CIM-496 showed symptoms of CLCuD at 11–14 days after germination. Grafts of BC_1_ from cross 2(*G. arboreum*)**×** *G. hirsutum* remained asymptomatic to this disease throughout their lifecycles, whereas only two grafts from BC_1_ of *G. hirsutum* **×** *G. arboreum* showed minor spots (3–5) of vein thickening at 40 DAG, which appeared on a few leaves. These minor spots become quite small at 70 DAG and were only detected after careful observation. Therefore, the BC_1_ hybrid plants of 2(*G. arboreum*)**×** *G. hirsutum* and *G. hirsutum* **×** *G. arboreum* were resistant to CLCuD, with good plant growth. The BC_2_ hybrid plants of 2(*G. arboreum*)**×** *G. hirsutum* and *G. hirsutum*×*G. arboreum* developed disease symptoms at 30–35 and 28–30 DAG, respectively. The infection type range for 2(*G. arboreum*)×*G. hirsutum* and *G. hirsutum*×*G. arboreum* was 0–1 and 0–2, respectively, and the same trend for the first appearance of disease symptoms was observed for BC_3_ plants from both crosses. All BC_3_ progenies from 2(*G. arboreum*)×*G. hirsutum* and *G. hirsutum*×*G. arboreum* were highly tolerant to CLCuD, with good fruit bearing and normal growth compared with susceptible variety CIM-496. By and large, the hybrid plants of cross 2(*G. arboreum*)×*G. hirsutum* showed better resistance/tolerance to CLCuD than those of cross *G. hirsutum*×*G. arboreum*.

**Table 6:** Evaluation of plants from an interspecific cross between *G. arboreum* and *G. hirsutum* against cotton leaf curl virus disease through grafting.

	Progeny	Year	No. ofplantstested	Graftingsuccess(%)	Infectivity(%)	Latentperiod(days)	Infectiontyperange^A^	Avdiseaseseverityafter 70(DAG)	Diseasereaction
BC_1_	[2(*G. arboreum*)×*G. hirsutum*]	2011	15	100	100	Symptomless	0	0	Resistant
	*G. hirsutum*×*G. arboreum*	2011	12	100	100	39–41	0–1	1	Highly tolerant
	CIM-496 (Std.)	2011	20	100	100	14	3–4E*	4E	Highly susceptible
BC_2_	[2(*G. arboreum*)×*G. hirsutum*]	2012	14	100	100	30–35	0–1	1	Highly tolerant
	*G. hirsutum*×*G. arboreum*	2012	20	100	100	28–30	0–2	1	Highly tolerant
	CIM-496 (Std.)	2012	10	100	100	11	3–4E	4E	Highly susceptible
BC_3_	[2(*G. arboreum*)×*G. hirsutum*]	2013	12	100	100	28–30	0–2	1	Highly tolerant
	*G. hirsutum*×*G. arboreum*	2013	16	100	100	25–30	0–3	1	Highly tolerant
	CIM-496 (Std.)	2013	10	100	100	11	3–4E	4E	Highly susceptible

A Infection type range is based on the 0–4 scale described in Table 1,

*Enation where observed.

### Testing of BC_1_ to BC_3_ progenies against CLCuD under natural field conditions

The BC_1_ to BC_3_ hybrid plants of 2(*G. arboreum*)×*G. hirsutum* and *G. hirsutum*×*G. arboreum* were tested under natural field conditions. Nineteen plants of BC_1_ of the combination [2(*G. arboreum*)×*G. hirsutum*] and 15 plants of reciprocal cross *G. hirsutum*×*G. arboreum* revealed disease indices of 1.3% and 1.6%, respectively, whereas the average severity index was 0.05 and 0.06 at 40 DAS, respectively (Table 7). However, the disease index and severity index were zero after 70 DAS because the minor spots of vein thickening that were observed on a single plant of each cross disappeared after 70 DAS. CIM 496, the control variety used in this trial, had a disease index of 94.3%, and enation was also observed at 70 DAS. Fourteen plants of BC_2_ of the combination 2(*G. arboreum*)×*G. hirsutum* and 161 plants of the combination *G. hirsutum*×*G. arboreum* raised through backcrossing of BC_1_ with *G. hirsutum* had disease indices of 1.8% and 4.0%, respectively, at 40 DAS, and the disease index increased to 3.5% and 6.8%, respectively, at 70 DAS. The grade of disease severity in 2(*G. arboreum*)×*G. hirsutum* was 0.07 (40 DAS) to 0.1 (70 DAS), whereas it was 0.17 (40 DAS) to 0.2 (70 DAS) for *G. hirsutum*×*G. arboreum*. The susceptible cotton variety CIM 496 in this trial had a disease index of 97.7% with a disease severity grade of 3.9.

**Table 7:** Evaluation of plants from an interspecific cross between *G. arboreum* and *G. hirsutum* against cotton leaf curl virus disease under natural field conditions.

Parentage		No. ofplantstested	Latentperiod(days)	No. of virusinfected plants		Diseaseindex(%)		Severityindex		Infectiontyperange^A^		Diseasereaction#
				40 DAS	70 DAS	40DAS	70DAS	40DAS	70DAS	40DAS	70DAS	
**BC_1_**	[2(*G. arboreum*)×*G. hirsutum*]	19	Symptomless	19(18^0^+1^1^)	19(19^0^)	1.3	0	0.05	0	0	0	R
	(*G. hirsutum*×*G. arboreum*)	15	35–40	15(14^0^+1^1^)	15(15^0^)	1.6	0	0.06	0	0–1	0	R
	CIM-496 (Std.)	31	14	31(1^2^+5^3^+25^4^)	31(4^3^+27^4^)	94.35	96.7E*	3.7	3.9	2–4E	3–4E	HS
**BC_2_**	[2(*G. arboreum*)×*G. hirsutum*]	14	30–35	14(13^0^+1^1^)	14(12^0^+2^1^)	1.8	3.5	0.07	0.1	0–1	0–1	HT
	*G. hirsutum*×*G. arboreum*	161	25–30	161(146^0^+7^1^+5^2^+3^3^)	161(138^0^+9^1^+8^2^+5^3^+1^4^)	4	6.8	0.17	0.2	0–3	0–4	HT
	CIM-496 (Std.)	168	13	168(4^2^+7^3^+157^4E^)	168(4^2^+11^3^+153^4E^)	97.7	97.1	3.9	3.9	2–4E	2–4E	HS
**BC_3_**	[2(*G. arboreum*)×*G. hirsutum*]	12	25–30	12(11^0^+1^2^)	12(9^0^+2^1^+1^2^)	4.2	8.3	0.17	0.3	0–2	0–2	HT
	*G. hirsutum*×*G. arboreum*	225	25–30	225(200^0^+5^1^+8^2^+6^3^+6^4^)	225(176^0^+14^1^+12^2^+11^3^+9^4^)	7	12	0.28	0.5	0–4	0–4	HT
	CIM-496 (Std.)	190	15	190(4^2^+6^3^+180^4E^)	190(6^2^+15^3^+169^4E^)	95	96.4	3.8	3.8	2–4E	2–4E	HS

A Infection type range is based on the 0–4 scale described in Table 1;

*Enation where observed; R Resistant; HT Highly tolerant; HS Highly susceptible;

# Disease reaction based on disease index 70 DAS.

Twelve plants of BC_3_ of the combination 2(*G. arboreum*)×*G. hirsutum* and 225 plants of the combination *G. hirsutum*×*G. arboreum* raised through backcrossing with *G. hirsutum* had a 4.2% and 7.0% disease index, respectively, at 40 DAS. And the disease index increased to 8.3% and 12.0%, respectively, at 70 DAS. The grade of disease severity in 2(*G. arboreum*)×*G. hirsutum* was 0.17 (40 DAS) to 0.3 (70 DAS), whereas it was 0.28 (40 DAS) to 0.5 (70 DAS) for *G. hirsutum*×*G. arboreum*. CIM 496 had a disease index of 95.0% with a disease severity grade of 3.8 (Table 7).

**Figure 8:**
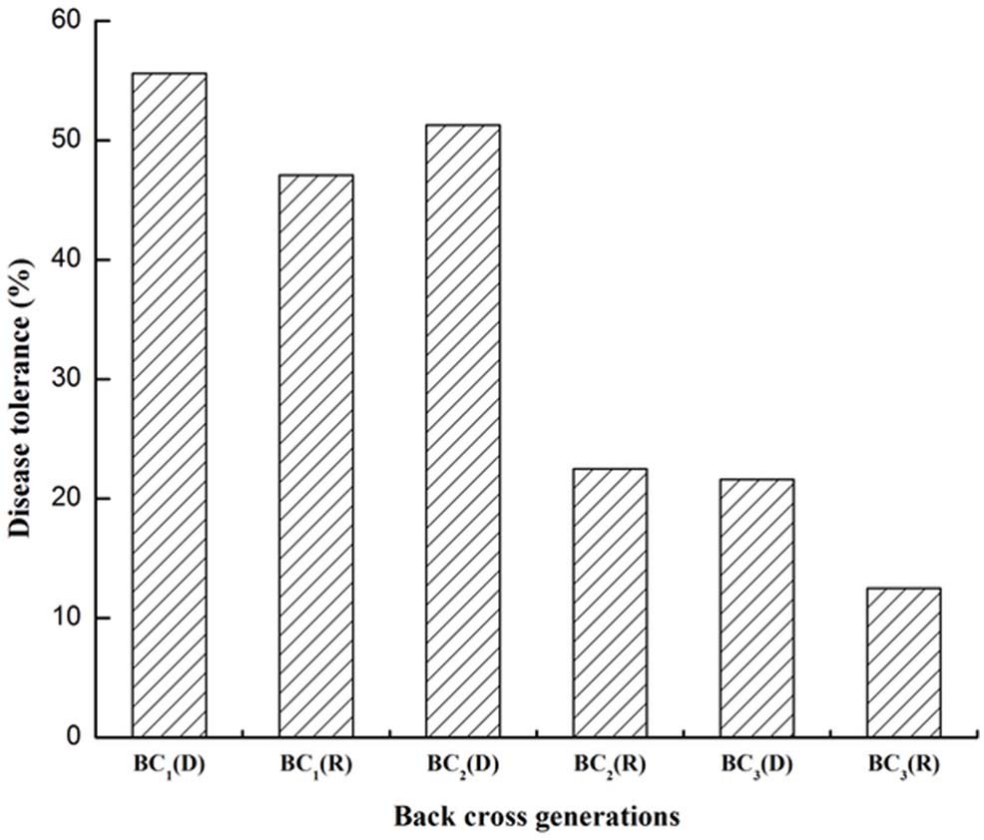
Percent disease tolerance across different generations (BC_1_ to BC_3_). D. Direct cross [2(*G. arboreum*)×*G. hirsutum*]; R. Reciprocal cross [*G. hirsutum*×*G. arboreum*].

As the backcross progressed from BC_1_ to BC_3_, the PDT gradually decreased (Figure 8). However, the PDT was fairly high in 2(*G. arboreum*)×*G. hirsutum* compared to the combination *G. hirsutum*×*G. arboreum*.

## Discussion

*G. hirsutum* has low genetic diversity and lacks resistance against CLCuD. In general, wild diploid species of *Gossypium* possess resistance against many challenges, such as insects, pests, diseases, and many abiotic factors [37], [46]. Hence, there is a great need to exploit this resource to develop resistance against CLCuD in cultivated tetraploid species [2]. Cotton breeders have long tried to obtain hybrids between diploid and tetraploid species [47]. However, several incompatibility factors hinder the development of hybrids under *in situ* conditions [48], [49]. Abortion of the embryo after fertilization and the lack of retention of cross bolls [50], [51] is a common stumble in interspecific crosses. Some species like *G. barbadense* can be hybridize easily with *G. hirsutum* and produce fertile F_1_ progeny [52] without hormones application. These two species i.e. *G. hirsutum* and *G. barbadense* have chromosome homology and the tetraploid genomes, are not separated by any large scale chromosomal rearrangement [53]. However, crosses between *G. hirsutum* and *G. arboreum* L. are rarely successful without hormone application [44], [54]. Plant hormones are known to control pollen tube growth [55]. Exogenous application of growth hormones has been used to overcome the crossing barrier and to facilitate interspecific crosses in many crops, i.e., cotton [56], wheat [57], and tomato [58]. Altman [56] compared exogenous application with *in vitro* techniques, i.e., ovule and embryo culture, and found that exogenous hormone application in conjunction with standard hybridization methods is superior to *in vitro* methods. Interspecific hybridization of cotton is enhanced by the application of exogenous hormones after pollination. Exogenous hormone application alone may be used to overcome certain crossing barriers within *Gossypium* [59]–[60]. The extract of garlic acid has been used as a growth regulator to obtain interspecific hybrids between tetraploid *G. hirsutum* and diploid *G. arboreum* species of cotton [56], [61]. The *in situ* development of BC_1_ to BC_3_ plants using exogenous hormones in the current study was superior to that using *in vitro* methods, which is in agreement with an earlier report [55]. The average number of seeds per boll varied from immature seeds to 1.5 seeds per boll. In the absence of exogenous hormones, pollinated flowers produce 0.1% seed development [56].

The boll setting and seed germiantion is very low in interspecific crosses and fertility of interspecific crosses can be measured in terms of boll setting percentage [52], [62]. The cross fertility of BC_1_ to BC_3_ between [2(*G. arboreum*)×*G. hirsutum*] and *G. hirsutum*×*G. arboreum* showed that the boll set was maximum (42.9%) in cross BC_3_, *G. hirsutum*×*G. arboreum*, but minimum (1.3%) in cross BC_2_, 2(*G. arboreum*)×*G. hirsutum* (Table 2). Viable seeds were obtained in both combinations. From BC_1_ to BC_3_, an increasing trend of boll setting and germination (%) was observed. Seed setting improvement was also recorded in *Brassica* by backcrossing with the recurrent parent [63]. The factor responsible for the semi-sterile condition are transmitted rarely through the pollen but readily through the egg cell. Boll setting and germination (%) was higher in reciprocal cross (*G. hirsutum*×*G.arboreum*) as compared to direct cross 2(*G. arboreum*)×*G. hirsutum* [64].

In general, the BC_1_ to BC_3_ hybrid plants of both cross combinations [2(*G. arboreum*)×*G. hirsutum* and *G. hirsutum*×*G. arboreum*] were intermediate in several traits between the two parents. The prevalence of yellow pollen in both crosses (direct and reciprocal) in most of the plants validated the inheritance of this character from *G. arboreum*, because this color is more common in *G. arboreum* species [65], [66], and it revealed the dominance in inheritance [67]. By contrast, in BC_1_ to BC_3_, leaf hairiness, flower size, corolla color, petal spots, pollen color, and so on were segregated in both parents. Moreover, morphological characteristics particularly leaf texture, leaf size, bracteole size, corolla color and petal spots from *G. arboreum* into BC_1_ to BC_3_ progenies [68] of both crosses were helpful for selection of plant progenies that have resemblance to *G. arboreum* and also showed CLCuD resistance. The frequency of plant progenies that showed good plant architecture were higher in reciprocal crosses as compared to direct cross.

When developing interspecific hybrids for resistance, a thorough knowledge of the chromosomal behavior in hybrids and backcross progenies is essential. In the present study, in hybrid 2(*G. arboreum*)×*G. hirsutum*, the ‘AD’ genome was introgressed into the ‘AA’ genome of *G. arboreum*, producing an ‘AAAD’ genomic constitution. In hybrid *G. hirsutum*×*G. arboreum*, the A-genome of *G. arboreum* was introgressed into the ‘AD’ genome of *G. hirsutum,* producing the genomic constitution ‘AAD’. In *G. arboreum* and *G. hirsutum*, normal orientation, association, and disjunction of chromosomes were observed, while in F_1_ hybrids of the above genomic constitution, quadrivalents and a low frequency of chromosome association (bivalents) were observed. The univalents observed in this study can be attributed to asynapsis due to the lack of homology between the different sets of chromosomes. The presence of laggards demonstrates the occurrence of meiotic disturbances, leading to an imbalance in the daughter cells. In BC_1_ hybrid plants of both combinations, the frequency of univalents and multivalents was high, and the plants were sterile/partially fertile. In BC_2_ hybrids of both combinations, the frequency of univalents and multivalents was low, and the plants were shy bearing. In BC_3_ hybrids of both combinations, the frequency of chromosome association (bivalents) was 25–26; hence, the plants were fertile. The average of univalent (I’s) chromosomes was higher in 2(*G. arboreum*)×*G. hirsutum* in comparison with *G. hirsutum*×*G.arboreum*. However, the average of bivalents (II’s) chromosomes was higher in *G. hirsutum*×*G.arboreum*. Thus *G. hirsutum*×*G.arboreum* was more fertile and more adaptive to the environment than 2(*G.arboreum*)×*G. hirsutum.*

Studies of resistance/susceptibility are rather difficult and laborious due to the involvement of vectors, the efficiency of transmission, and the persistent nature of the virus/CLCuD. Grafting may successfully lead to the transmission of the virus when other methods fail, as it involves the union of cambial layers of the root sock and scion [69]–[70]. Thus, to screen CLCuD-resistant germplasm, transmission by grafting is the best alternative to natural transmission by vector, as most viruses of a persistent nature, such as CLCuD, cannot be transmitted through mechanical inoculation [71]. Ahmad et al. [72] used sick plot techniques to screen the exotic and local germplasm against CLCuD.

The BC_1_ to BC_3_ of 2(*G. arboreum*)×*G. hirsutum* and *G. hirsutum*×*G. arboreum* were tested through grafting under natural field/greenhouse conditions. These hybrids remained resistant to CLCuD [39]. The results of evaluation of the BC_1_ to BC_3_ progenies revealed a high degree of variability for CLCuD in the field and through grafting. All plants from both crosses [2(*G. arboreum*)×*G. hirsutum* and *G. hirsutum*×*G. arboreum*] showed 100% infectivity and grafting success. However, latent period and infection type range for BC_1_–BC_2_ was better in 2(*G.arboreum)*×*G. hirsutum* cross than *G. hirsutum*×*G.arboreum.* The grafts of BC_1_ from cross 2(*G. arboreum*)×*G. hirsutum* remained asymptomatic to this disease. However, BC_1_ of *G. hirsutum*×*G. arboreum* showed minor vein thickening, but the vein thickening was highly reduced after 70 days of grafting [46], whereas CIM-496 showed symptoms of CLCuD within 11–14 days after germination. Although minor symptoms of CLCuD appeared in BC_1_ of *G. hirsutum*×*G. arboreum*, this disease did not affect the growth of the plants. Therefore, we can conclude that these plants were also resistant to CLCuD. The BC_2_ and BC_3_ hybrid plants of both cross combinations 2(*G. arboreum*)×*G. hirsutum* and *G. hirsutum*×*G. arboreum* developed disease symptoms after 28–35 DAG, and the average disease severity was grade 1.0 (70 DAG). Additionally, these plants showed good tolerance to CLCuD, with no symptoms of stunted growth. Therefore, these BC_2_ and BC_3_ plants were highly tolerant to CLCuD compared with susceptible variety CIM-496, which showed CLCuD symptoms after 11 DAS with no boll setting. Ullah et al. [46] also observed mild symptoms of CLCuD on the introgressed material following grafting, but the amount of viral DNA was significantly lower than the levels found in *G. hirsutum.* The same trend/response for latent period to acquire CLCuD was observed in the field for BC_1_ to BC_3_ hybrid plants. The average severity index for 2(*G. arboreum*)×*G. hirsutum* and *G. hirsutum*×*G. arboreum* was 0.05 and 0.06 (40 DAS), respectively. However, the disease index and severity index were zero after 70 DAS. Thus, the resistant hybrid plants of both crosses 2(*G. arboreum*)×*G. hirsutum* and *G. hirsutum*×*G. arboreum* showed better tolerance to CLCuD, with not deleterious effects on yield or growth. However, 2(*G. arboreum*)×*G. hirsutum* plants were more tolerant regarding number of virus infected plants, disease index (%), severity index and infection type range than those of cross *G. hirsutum*×*G. arboreum.* Collectively, plants from these crosses had better tolerance to CLCuD than CIM-496. The PDT was higher in 2(*G. arboreum*)×*G. hirsutum* than in *G. hirsutum*×*G. arboreum*. The frequency of ideotype plants was higher in *G. hirsutum*×*G. arboreum* compared with 2(*G. arboreum)*×*G. hirsutum.*

## Conclusions

The results indicate that the BC_1_ to BC_3_ progenies were highly tolerant to CLCuD, indicating the possibility of transferring CLCuD resistance genes from *G. arboreum* to *G. hirsutum* through conventional hybridization and backcrossing. As the backcross progressed, the disease incidence also increased, from BC_1_ (1.3–1.6%) to BC_2_ (1.8–4.0%) to BC_3_ (4.2–7.0%). However, the disease incidence was much lower than that of the commercial cultivar CIM-496, which exhibited a very high incidence of CLCuD (97.7%). The disease incidence was lower in combination 2(*G. arboreum*)×*G. hirsutum* than in *G. hirsutum*×*G. arboreum*. As “A” genome is an invaluable genetic resource for improving modern tetraploid cotton (*G. hirsutum*). We observed very wide genetic variability among BC_1_ to BC_3_ progenies, which will certainly facilitate improvement of cotton resistances to diseases. And various scientists also utilized *G. arboreum* L. for introgression of desirable resistant genes into cultivated tetraploid cotton for drought [34]–[35], heat [36], root rot, cotton leaf curl virus [37,[44] and insect pests (bollworms and aphids) [12]. Therefore, the introgression lines of *G. arboreum* developed with or wothout resistance in this study can be employed to map the resistance gene(s)/loci, which will be very useful for future diverse (a)biotic-tolerant cotton breeding.

## References

[1] Tiendrébéogo F, Lefeuvre P, Hoareau M, Villemot J, Konaté G, et al (2010) Molecular diversity of Cotton leaf curl Gezira virus isolates and their satellite DNAs associated with okra leaf curl disease in Burkina Faso. Virology Journal 7: 48. 20178575 10.1186/1743-422X-7-48 PMC2839976

[2] Nazeer W, Ahmad S, Mahmood K, Tipu A, Mahmood A, et al (2014) Introgression of genes for cotton leaf curl virus resistance and increased fiber strength from Gossypium stocksii into upland cotton (Gossypium hirsutum). Genetics and molecular research 13: 1133–1143. 24634169 10.4238/2014.February.21.2

[3] Rajagopalan PA, Naik A, Katturi P, Kurulekar M, Kankanallu RS, et al (2012) Dominance of resistance-breaking cotton leaf curl Burewala virus (CLCuBuV) in northwestern India. Archives of virology 157: 855–868. 22307170 10.1007/s00705-012-1225-y

[4] Tahir MN, Amin I, Briddon RW, Mansoor S (2011) The merging of two dynasties–identification of an African cotton leaf curl disease-associated begomovirus with cotton in Pakistan. PloS One 6: e20366. 21637815 10.1371/journal.pone.0020366 PMC3102712

[5] Ahuja SL, Monga D, Dhayal LS (2007) Genetics of resistance to cotton leaf curl disease in Gossypium hirsutum L. under field conditions. Journal of heredity 98: 79–83. 17159229 10.1093/jhered/esl049

[6] Briddon RW (2003) Cotton leaf curl disease, a multicomponent begomovirus complex. Molecular Plant Pathology 4: 427–434. 20569402 10.1046/j.1364-3703.2003.00188.x

[7] Mansoor S, Zafar Y, Briddon RW (2006) Geminivirus disease complexes: the threat is spreading. Trends in plant science 11: 209–212. 16616578 10.1016/j.tplants.2006.03.003

[8] Azhar MT, Amin I, Anjum ZI, Arshad M, Briddon RW, et al (2010) Both malvaceous and non-malvaceous betasatellites are associated with two wild cotton species grown under field conditions in Pakistan. Virus genes 41: 417–424. 20798983 10.1007/s11262-010-0521-4

[9] Hussain T, Ali M (1975) A review of cotton diseases of Pakistan. Pakistan Cottons 19: 71–86.

[10] Hussain T, Mahmood T (1988) A note on leaf curl disease of cotton. Pakistan Cotton 32: 248–251.

[11] Thakur P (2002) Virus diseases of cotton. Diseases of Field Crops: 398.

[12] Mansoor S, Amin I, Iram S, Hussain M, Zafar Y, et al (2003) Breakdown of resistance in cotton to cotton leaf curl disease in Pakistan. Plant pathology 52: 784–784.

[13] Zafar Y, Brown J (2011) Genome characterization of whitefly-transmitted geminivirus of cotton and development of virus-resistant plants through genetic engineering and conventional breeding. The ICAC Recorder 29: 7–12.

[14] Briddon R, Markham P (2000) Cotton leaf curl virus disease. Virus research 71: 151–159. 11137169 10.1016/s0168-1702(00)00195-7

[15] Mann R (2011) Bemisia tabaci Interaction with Cotton Leaf Curl Virus. In: Thompson WMO (ed) The Whitefly, Bemisia tabaci (Homoptera: Aleyrodidae) Interaction with Geminivirus-Infected Host Plants. Springer Netherlands, 69–88.

[16] Singh D, Gill J, Gumber R, Singh R, Singh S (2013) Yield and fibre quality associated with cotton leaf curl disease of Bt-cotton in Punjab. Journal of Environmental Biology 34: 113–116. 24006816

[17] Rahman M, Hussain D, Malik T, Zafar Y (2005) Genetics of resistance to cotton leaf curl disease in Gossypium hirsutum. Plant pathology 54: 764–772.

[18] Ahmad S, Hussain A, Hanif M, Mahmood K, Nazeer W, et al (2012) CRSM-38, a new high yielding coupled with CLCuV tolerance cotton (Gossypium hirsutum L.) variety. African Journal of Biotechnology 11: 4368–4677.

[19] Mahmood T, Arshad M, Gill MI, Mahmood HT, Tahir M, et al (2003) Burewala strain of cotton leaf cur l virus: A threat to CLCuV cotton resistance varieties. Asian Journal Plant Sciences 2: 968–970.

[20] Tahir M, Tariq M, Mahmood H, Hussain S (2004) Effect of sowing dates on incidence of cotton leaf curl virus on different cultivars of cotton. Plant Pathology Journal 3: 61–64.

[21] Amrao L, Akhter S, Tahir MN, Amin I, Briddon RW, et al (2010) Cotton leaf curl disease in Sindh province of Pakistan is associated with recombinant begomovirus components. Virus research 153: 161–165. 20621137 10.1016/j.virusres.2010.07.003

[22] Akhtar K, Haidar S, Khan M, Ahmad M, Sarwar N, et al (2010) Evaluation of Gossypium species for resistance to cotton leaf curl Burewala virus. Annals of Applied Biology 157: 135–147.

[23] Mahmood T, Arshad M, Gill MI, Mahmood HT, Tahir M, et al (2003) Burewala strain of cotton leaf curl virus: a threat to CLCuV cotton resistant varieties. Asian Journal of Plant Sciences 2: 968–970.

[24] Cai J, Xie K, Lin L, Qin B, Chen B, et al (2010) Cotton leaf curl Multan virus newly reported to be associated with cotton leaf curl disease in China. Plant pathology 59: 794–795.

[25] Sattar MN, Kvarnheden A, Saeed M, Briddon RW (2013) Cotton leaf curl disease–an emerging threat to cotton production worldwide. Journal of General Virology 94: 695–710. 23324471 10.1099/vir.0.049627-0

[26] Azhar MT, Akhtar S, Mansoor S (2012) Letter to the Editor: Cotton leaf curl Multan betasatellite strains cloned from Gossypium barbadense further supports selection due to host resistance. Virus genes 45: 402–405. 22644763 10.1007/s11262-012-0766-1

[27] Farooq A, Farooq J, Mahmood A, Shakeel A, Rehman A, et al (2011) An overview of cotton leaf curl virus disease (CLCuD) a serious threat to cotton productivity. Australian Journal of Crop Science 5: 1823–1831.

[28] Zaffalon V, Mukherjee SK, Reddy VS, Thompson JR, Tepfer M (2012) A survey of geminiviruses and associated satellite DNAs in the cotton-growing areas of northwestern India. Archives of virology 157: 483–495. 22209785 10.1007/s00705-011-1201-y

[29] Amin K (1940) Interspecific hybridization between Asiatic and New World cottons. Indian Journal of Agricultural Science 10: 404–413.

[30] Blank L, Lathers C (1963) Environmental and other factors influencing development of south western cotton rust. Phytopatholgy 53: 921–928.

[31] Nelson RR (1973) Breeding plants for disease resistance concepts and applications. University Park, Penn.: The Pennsylvania State University Press.

[32] Kalloo G (1992) Utilization of Wild Species. In: Kalloo G, Chowdhury JB, editors. Distant Hybridization of Crop Plants: Springer Berlin Heidelberg. 149–167.

[33] Azhar MT, Aftab S, Zafar Y, Mansoor S (2010) Utilization of natural and genetically-engineered sources in Gossypium hirsutum for the development of tolerance against cotton leaf curl disease and fiber characteristics. International Journal of Agriculture and Biollogy 12: 744–748.

[34] Maqbool A, Abbas W, Rao AQ, Irfan M, Zahur M, et al (2010) Gossypium arboreum GHSP26 enhances drought tolerance in Gossypium hirsutum. Biotechnology Progress 26: 21–25. 19847887 10.1002/btpr.306

[35] Zhang L, Li FG, Liu CL, Zhang CJ, Zhang XY (2009) Construction and analysis of cotton (Gossypium arboreum L.) drought-related cDNA library. BMC research notes 2: 120. 19570239 10.1186/1756-0500-2-120 PMC2714314

[36] Zahur M, Maqbool A, Irfan M, Barozai MYK, Qaiser U, et al (2009) Functional analysis of cotton small heat shock protein promoter region in response to abiotic stresses in tobacco using Agrobacterium-mediated transient assay. Molecular Biology Reports 36: 1915–1921. 18991019 10.1007/s11033-008-9399-9

[37] Azhar M, Anjum Z, Mansoor S (2013) Gossypium gossypioides: A source of resistance against cotton leaf curl disease among D genome diploid cotton species. JAPS, Journal of Animal and Plant Sciences 23: 1436–1440.

[38] Cao Z, Wang P, Zhu X, Chen H, Zhang T (2013) SSR marker-assisted improvement of fiber qualities in Gossypium hirsutum using Gossypium barbadense introgression lines. Theoretical and Applied Genetics: 1–8. 10.1007/s00122-013-2241-3 24306319

[39] Ahmad S, Khan N, Mahmood A, Mahmood K, Sheikh AL, et al.. (2011) Exploring potential sources for leaf curl virus resistance in cotton (Gossypium hirsutum L.). 5th meeting of Asian Cotton Research and Development network Lahore Pakistan Lahore, Pakistan: International Cotton Advisory Committee (ICAC).

[40] Guo W, Wang W, Zhou B, Zhang T (2006) Cross-species transferability of G. arboreum-derived EST-SSRs in the diploid species of Gossypium. Theoretical and Applied Genetics 112: 1573–1581. 16596396 10.1007/s00122-006-0261-y

[41] Sacks EJ, Robinson AF (2009) Introgression of resistance to reniform nematode (Rotylenchulus reniformis) into upland cotton (Gossypium hirsutum) from Gossypium arboreum and a Gossypium hirsutum/Gossypium aridum bridging line. Field Crops Research 112: 1–6.

[42] Chen Y, Wang Y, Wang K, Zhu X, Guo W, et al (2014) Construction of a complete set of alien chromosome addition lines from Gossypium australe in Gossypium hirsutum: morphological, cytological, and genotypic characterization. Theoretical and Applied Genetics 127: 1105–1121. 24553965 10.1007/s00122-014-2283-1 PMC3997835

[43] Mergcai G, Baudoin JP, Vroh Bi I (1997) Exploitation of trispecific hybrids to introgress the glandless seed and glanded plant trait of Gossypium sturtianum Willis into Gossypium hirsutum L. Biotechnologie Agronomie Societe et Environment. 1: 272–277.

[44] Ahmad S, Mahmood K, Hanif M, Nazeer W, Malik W, et al (2011) Introgression of cotton leaf curl virus-resistant genes from Asiatic cotton (Gossypium arboreum) into upland cotton (G. hirsutum). Genetics and Molecular Research 10: 2404–2414. 22002133 10.4238/2011.October.7.2

[45] Akhtar KP, Khan AI, Hussain M, Khan MSI (2002) Comparison of resistance level to cotton leaf curl virus(CLCuV) among newly developed cotton mutants and commercial cultivars. Plant Pathollogy Journal 18: 179–186.

[46] Ullah R, Akhtar KP, Moffett P, Mansoor S, Briddon RW, et al.. (2014) An analysis of the resistance of Gossypium arboreum to cotton leaf curl disease by grafting. European Journal of Plant Pathology: 1–11.

[47] Gill MS, Bajaj Y (1987) Hybridization between diploid (Gossypium arboreum) and tetraploid (Gossypium hirsutum) cotton through ovule culture. Euphytica 36: 625–630.

[48] Sikka S, Joshi A (1960) Breeding. Cotton in India-a monograph Indian Central Cotton Committee, Bombay: 137–235.

[49] Thengane S, Paranjpe S, Khuspe S, Mascarenhas A (1986) Hybridization of Gossypium species through in ovulo embryo culture. Plant cell, tissue and organ culture 6: 209–219.

[50] Borole V, Dhumale D, Rajput J (2000) Embryo culture studies in interspecific crosses between arboreum and hirsutum cotton. Indian Journal of Genetics and Plant Breeding 60: 105–110.

[51] Pundir N (1972) Experimental embryology of Gossypium arboreum L. and Gossypium hirsutum L. and their reciprocal crosses. Botanical gazette: 7–26.

[52] Brubaker C, Brown A, Stewart JM, Kilby M, Grace J (1999) Production of fertile hybrid germplasm with diploid Australian Gossypium species for cotton improvement. Euphytica 108: 199–214.

[53] Gerstel D, Sarvella PA (1956) Additional observations on chromosomal translocations in cotton hybrids. Evolution: 408–414.

[54] Jafari Mofidabadi A, Soltanloo H, Ranjbran A (2011) Development of Genetic Broadening System in Cotton through Artificial Crosses between 2x and 4x Species. Cotton Genomics and Genetics 2.

[55] Kovaleva L, Zakharova E, Minkina YV, Timofeeva G, Andreev I (2005) Germination and in vitro growth of petunia male gametophyte are affected by exogenous hormones and involve the changes in the endogenous hormone level. Russian Journal of Plant Physiology 52: 521–526.

[56] Altman D (1988) Exogenous hormone applications at pollination for in vitro and in vivo production of cotton interspecific hybrids Plant Cell Reports. 7: 257–261. 10.1007/BF00272537 24241761

[57] Sitch L, Snape J (1987) Factors affecting haploid production in wheat using the Hordeum bulbosum system. 1. Genotypic and environmental effects on pollen grain germination, pollen tube growth and the frequency of fertilization. Euphytica 36: 483–496.

[58] Gordillo L, Jolley V, Horrocks R, Stevens M (2003) Interactions of BA, GA3, NAA, and surfactant on interspecific hybridization of Lycopersicon esculentum×Lycopersicon chilense. Euphytica 131: 15–23.

[59] Liang Z, Sun C (1982) The significant effect of endosperm development on the interspecific hybridization of cotton. ActaGenetSinica (in Chinese) 9: 441–454.

[60] Liang CL, Sun CW, Liu TL, Chiang JC (1978) Studies on interspecific hybridization in cotton. Scientia Sinica 21: 545–555.

[61] Mofidabadi A (2009) Producing triploid hybrids plants through induce mutation to broaden genetic base in cotton. The ICAC Recorder 27: 10–11.

[62] Jorgensen RB, Andersen B (1994) Spontaneous hybridization between oilseed rape (Brassica napus) and weedy B. campestris (Brassicaceae): a risk of growing genetically modified oilseed rape. American Journal of Botany: 1620–1626.

[63] Roy N (1980) Species crossability and early generation plant fertility in interspecific crosses of Brassica. Sabrao Journal 12: 43–53.

[64] Ali M, Lewis C (1962) Effects of Reciprocal Crossing on Cytological and Morphological Features of Interspecific Hybrids of Gossypium hirsutum L. and G. barbadense L. Crop Science. 2: 20–22.

[65] Stephens S (1954) Interspecific homologies between gene loci in Gossypium. I. Pollen color. Genetics 39: 701–711. 17247514 10.1093/genetics/39.5.701 PMC1209683

[66] Silow RA (1941) The comparative genetics of Gossypium anomalum and the cultivated Asiatic cottons. Journal of Genetics 42: 259–358.

[67] Harland SC (1929) The genetics of cotton. Part II. The inheritance of pollen colour in New World cottons. Journal of Genetics 20: 387–399.

[68] Deshpande L, Kokate R, Kulkarni U, Nerkar Y (1991) Cytomorphological studies in induced tetraploid G. arboreum and its interspecific hybrid with tetraploid G. hirsutum L. The Indian Journal of Genetics and Plant Breeding. 51: 194–202.

[69] Matthews R (1991) Plant virology: Elsevier, San Diego:Academic Press, Inc.

[70] Akhtar K, Khan A, Hussain M, Haq M, Khan M (2003) Upland cotton varietal response to cotton leaf curl virus (CLCuV). Tropical Agricultural Research & Extension 5: 29–34.

[71] Akhtar KP, Khan MSI, Khan AI (2002) Improved bottle shoot grafting technique/method for the transmission of cotton leaf curl virus (CLCuV). FAO. Available: http://agris.fao.org/agris-search/search.do?recordID=PK2004000384#. 115–117.

[72] Ahmad S, Khan N, Mahmood A, Nazeer W, Ahmad S, et al (2011) Screening of cotton germplasm against cotton leaf curl virus. Pakistan Journal Botany 43: 725.

